# Causal Association Between Tea Consumption and Bone Health: A Mendelian Randomization Study

**DOI:** 10.3389/fnut.2022.872451

**Published:** 2022-04-26

**Authors:** Song Chen, Tianlai Chen, Yibin Chen, Dianhua Huang, Yuancheng Pan, Shunyou Chen

**Affiliations:** ^1^Department of Orthopedics, Fuzhou Second Hospital, Fuzhou, China; ^2^The Third Department of Clinical Medicine, Fujian Medical University, Fuzhou, China; ^3^Fujian University of Traditional Chinese Medicine, Fuzhou, China

**Keywords:** tea consumption, osteoarthritis, rheumatoid arthritis, osteoporosis, SNP, mendelian randomization

## Abstract

**Background:**

Much observational research reported that tea consumption decreases the risk of osteoarthritis (OA), rheumatoid arthritis (RA), and osteoporosis (OP) which are the three major bone disorders. However, the observed correlation is inconclusive. To determine the causal relationship between genetically predicted tea intake and OA, RA, and OP, we performed a two-sample Mendelian randomization (MR) study based on large samples.

**Methods:**

The European population’s genome-wide association meta-analysis (GWAS) dataset identified SNPs associated with tea consumption was obtained from Neale Lab’s analysis of UK Biobank data that comprised 349,376 participants of European ancestry. We extracted genetic data for knee OA (17,885 controls and 4,462 cases), hip OA (50,898 controls and 12,625 cases), and RA (43,923 controls and 14,361 cases) from the UK Biobank and OP cases (93083 controls and 1,175 cases) from FinnGen Data Freeze 2. A MR study was conducted to examine the effect of selected single nucleotide polymorphisms (SNPs) and OA, RA, and OP risk. Several sensitivity analyses were performed with weighted median and inverse-variance weighted methods for estimating the causal effects.

**Results:**

In this MR study, the genetically predicted per one cup increase of tea consumption was not associated with knee OA (OR 1.11,95% CI: 0.79–1.55) using IVW with random effect. Genetic predisposition to tea consumption was not associated with hip OA (OR: 1.20, 95% CI: 0.84–1.71), RA (OR: 1.24 95% CI: 0.81–1.91), and OP (OR: 1.11, 95% CI: 0.89, 1.39). Following the sensitivity analysis, there was no potential pleiotropy.

**Conclusion:**

According to our study, According to our study, there was no statistical power to confirm a causal relationship between tea consumption and the risk of knee OA, hip OA, RA, and OP.

## Introduction

Chronic diseases and economic burdens increase as the population ages (1). Osteoarthritis (OA), Rheumatoid Arthritis (RA), osteoporosis (OP) are the three major bone disorders that can cause persistent chronic pain in the elderly (2). The disorder negatively impacts patients’ quality of life due to pain, depression, functional and social disabilities, and it can even impose social costs in severe cases (3–5). Research has explored many factors associated with these bone disorders, such as body mass index (BMI), coffee consumption, tea intake, etc (3, 4).

After water, tea is the most popular drink globally. Like the Mediterranean diet, all kinds of tea are rich in polyphenols, antioxidants that may reduce the risk of inflammatory diseases such as RA (6). As the world’s second most consumed beverage, tea contains flavonoids that may contribute to bone health. Studies show that habitual consumption of tea is positively associated with higher BMD at multiple skeleton sites (7). The use of green tea polyphenols may be beneficial to OA as a therapeutic addition by controlling IL-1β activity (8). There is evidence that tea consumption may benefit RA patients, suggesting that tea consumption could reduce disease activity (9). Green tea possesses powerful antioxidant and anti-inflammatory properties, which can counteract the harmful effects of OP, induced imbalances between osteoblastogenesis and osteoclastogenesis (10).

However, tea contains a lot of caffeine. There are many Mendelian randomization (MR) studies on the relationship between coffee and various types of arthritis. Habitual coffee consumption increases the incidence of OA (11), but not all joints. The effects of coffee were more prominent in knee OA, but not in the hip joint (12). Another Mendelian study found no causal effect of coffee consumption on the incidence of RA (13). Caffeine in tea release amount varies depending on the type of tea and the brewing method. Following 1 min of brewing in 194–203°F (90–95°C) water, a mug of Tazo Earl Gray contains 40 mg of caffeine (14). The caffeine in tea also contributes to the development of mechanical stress-induced OA, suggesting that tea could be a risk factor that promotes OA. Furthermore, Hashempur et al. found a positive relationship between incident RA and caffeinated tea consumption (15). For OP, A study of pre and perimenopausal women (50–60 years of age) in the United States found an inverse relationship between tea consumption and bone mineral density (16).

Since most studies on tea consumption and risk of OP, OA, and RA are observational or experimental, we are unsure whether the confounding factors or reverse causality attribute to the observed associations.

Mendelian randomization is a genetic epidemiological method, which uses genetic variants as instrumental variables. Reverse causation and potential confounding factors can be eliminated by MR (17). Single nucleotide polymorphism (SNPs) are assigned randomly at conception, avoiding residual confounding (18). The causal association between tea consumption with OA, RA, and OP has not been studied using MR. Therefore, we conducted a two-sample MR study to determine the causation.

## Materials and Methods

### Genetic Instrument Selection

According to Neale Lab genome-wide association meta-analysis (GWAS round 2),^1^ the GWAS summary statistics for tea intake (phenotype code: 1488_raw) were based on 349,376 samples of European ancestry from UK Biobank. This GWAS adjusted for age, sex, age 2, sex × age 2, sex × age, and the top twenty principal components. Habitual tea intake was retrieved based on the item: “How many cups of tea do you drink each day?” All instrumental variables were associated with the exposure (e.g., tea consumption) at a genome-wide significance level (*P* < 5 × 10^–8^) with linkage disequilibrium (LD) *r*^2^ < 0.01 at a 10,000 kb window, which confirmed the independence for the selected genetic variants. In this MR study, 22 independent SNPs with moderate LD were selected as genetic instruments for habitual tea consumption. Detailed information on the relationship between the selected SNPs and exposures is shown in Table 1.

**TABLE 1:** Characteristics of SNPs for habitual tea consumption from the GWAS.

SNP	chr	Pos	Closet gene	EAF	EA	OA	Effect	SE	*P*-value	*N*
rs6697410	1	26756209	LIN28A	0.75	T	G	0.0436	0.0079	4.10E-08	349376
rs199621380	1	150700614	CTSS	0.41	G	T	0.0413	0.0070	4.53E-09	349376
rs11487328	1	174601659	RABGAP1L	0.38	C	G	−0.0493	0.0071	5.16E-12	349376
rs1481012	4	89039082	ABCG2	0.11	G	A	−0.0778	0.0109	9.41E-13	349376
rs149375687	5	152034989	LINC01470	0.27	T	G	−0.0449	0.0078	7.26E-09	349376
rs2465018	6	51241140	RP11-22806.2	0.23	A	G	0.0634	0.0082	1.38E-14	349376
rs4410790	7	17284577	AC003075.4	0.64	C	T	0.1215	0.0072	1.89E-64	349376
rs73073176	7	17562952	AC017060.1	0.13	T	C	−0.0674	0.0103	5.78E-11	349376
rs3815455	7	75611756	POR	0.29	T	C	0.0647	0.0076	1.74E-17	349376
rs11022751	11	13307613	ARNTL	0.27	C	T	0.0497	0.0078	1.83E-10	349376
rs10741694	11	16286183	SOX6	0.63	C	T	0.0404	0.0071	1.53E-08	349376
rs1669433	12	11349732	RP11-144023.22	0.16	G	A	0.0551	0.0093	3.33E-09	349376
rs1601409	12	17066769	RP11-1018J11.1	0.47	G	A	0.0382	0.0069	3.67E-08	349376
rs7999399	13	89233505	RP11-360A9.3	0.56	T	C	0.0379	0.0069	4.96E-08	349376
rs12591786	15	60902512	RORA	0.16	T	C	−0.0609	0.0096	2.32E-10	349376
rs2472297	15	75027880	CYP1A1	0.26	T	C	0.1576	0.0078	3.82E-91	349376
rs12600469	17	40834073	CCR10	0.62	T	G	0.0406	0.0071	1.22E-08	349376
rs2315024	19	19423817	SUGP1	0.34	A	T	0.0434	0.0073	2.98E-09	349376
rs140775622	20	62962869	RP11-476I15.6	0.17	T	C	0.0707	0.0099	9.33E-13	349376
rs4817505	21	34343828	AP000282.2	0.39	C	T	0.0411	0.0071	6.22E-09	349376
rs9624470	22	24820268	ADORA2A	0.58	A	G	0.0729	0.0070	3.06E-25	349376
rs73424602	22	41461176	AL080243.1	0.40	T	C	−0.0432	0.0070	7.84E-10	349376

*Chr, chromosome; Pos, position for SNP; Closet gene, the nearest gene to coffee consumption associated SNP; Effect, the per-allele effect on tea consumption; P-value, the value for the genetic association; SNP, single-nucleotide polymorphism; EA, effect allele; OA, other allele; EAF, effect allele frequency (European); SE, standard error.*

### Genetic Summary Data of Osteoarthritis, Rheumatoid Arthritis, and Osteoporosis

The primary outcome in this study was the clinically diagnosed knee OA, hip OA, RA, and OP. The Summary data was available from a wide-ranging meta-analysis of transethnic GWAS. Over 100,000 participants of European and Asian descent participated in the survey. To reduce population stratification bias, we only analyzed data for European, including knee OA (17,885 controls and 4462 cases), hip OA (50,898 controls and 12,625 cases), and RA (43,923 controls and 14,361 cases) from the large GWAS. The secondary outcomes were specifically diagnosed OP. Data of OP was taken from the FinnGen Data Freeze 2.^2^ On January 14, 2020, FinnGen Data Freeze 2 was released to the public. It includes 96,499 individuals, 16 M variants, and 1,122 endpoints. The dataset contains 4,477 OP cases (93083 controls and 1175 cases). Study protocols related to these data have been released and described in the previous studies (19–21). All outcome summary GWAS data in this study were derived from ‘‘ieu open gwas project.^3^ “The GWAS IDs for the outcomes are as follows: ebi-a-GCST005813 for Knee OA, ebi-a-GCST005810 for hip OA, ieu-a-832 for RA, finn-b-M13_OSTEOPOROSIS for OP. The relevant ethics committees approved all studies that contributed data to these analyses, and all participants provided written informed consent.

### Statistical Analysis

**FIGURE 1:**
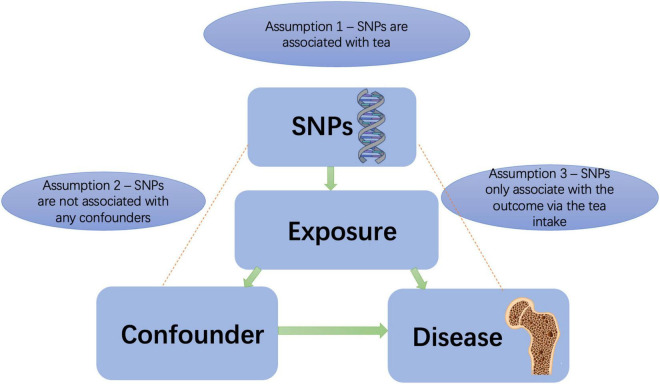
Principles of mendelian randomization study. SNPs, single nucleotide polymorphisms.

This was a two-sample MR analysis. MR, using genetic variants as instrumental variables, is a genetic epidemiologic method that can avoid potential confounding factors and reverse causation. It is assumed that the instrumental variables are valid if it meet all of the following criteria: (1) strongly associated with tea consumption, (2) not associated with confounding factors, and (3) risk association with knee OA, hip OA, RA, and OP were only detected *via* tea consumption (22, 23) (Figure 1). We tested for weak instrumental variables using *F* statistics. The F statistic equals [(n-k-1)/k) × R^2/(1-R^2)], where *R*^2^ represents the variance in tea consumption explained by the genetic instrument, K represents the number of genetic variations, and N represents the sample size. The *R*^2^ was calculated as follows: 2 × beta^2^ × EAF × (1-EAF)/2 × beta^2^ × EAF × (1-EAF) + se2 × 2 × N × EAF(1-EAF). EAF represents effect allele frequency (24). When *F* > 10, it is unlikely that a weak instrument will bias the MR estimates. Nevertheless, there may be pleiotropic genetic variants introduced, thus invalidating the IV.

The Cochran *Q*-test was applied to check SNPs’ statistical heterogeneity using MR-Egger estimates, with *P* < 0.05 deemed significantly heterogeneous. Therefore, the IVW method based on random effects was adopted. Several approaches were used to test whether the second and third MR assumptions were violated, including the MR-Egger regression (25), the weighted median (26) and mode (27) methods, an outlier test (MR-PRESSO) (28). MR-Egger is a weighted regression approach that introduces an intercept to accommodate pleiotropy. There was horizontal pleiotropy when the intercept term was away from zero (25). Using this approach, unbiased estimates are achieved in the presence of pleiotropic instruments with the assumption that the magnitude of pleiotropic effects cannot be predicted by the size of the instrumental variables—SNPs related to tea consumption (25).

In addition, funnel plots were used to assess pleiotropy. If the funnel plot is symmetric, there is no potential directional pleiotropy (29). We performed a “leave-one-out” analysis by excluding each SNP in turn. Therefore, individual SN*P* could be assessed for their influence on causal associations. The PhenoScanner database (version 2.0) was examined for each SNP (30) to determine whether existing risk factors have previously been associated with any significant associations (*P* < 5 × 10^–8^): BMI (31, 32), previous knee injury (32), gout (33), coffee intake (12) for knee OA; Hip dysplasia (34), Steroids use (35), coffee intake (11) for hip OA; hypertension (33), diabetes, smoking (36) for RA; Vitamin D intake, Steroids use, smoking for OP (37). To rule out potential pleiotropic effects, we evaluated the effect of removing these SNPs from the MR estimations.

All analyses were conducted using the “TwoSampleMR,” “MRPRESSO” R packages in RStudio version 3.6.3. The Bonferroni adjustment was used to correct for multiple comparisons (*P*-value: 0.05/4 outcomes = 0.0125). We computed two-side *P*-values, with *P* < 0.0125 regarded as statistically significant.

## Results

### Mendelian Randomization Study Design

All 22 SNPs together as the instrument together have an F-statistic more than 10 meeting the first assumption for MR study (Table 1). The PhenoScanner database showed one SNPs (rs10741694) associated with RA confounding factors (hypertension, diabetes, smoking), four other SNPs (rs11022751; rs1481012; rs2472297; rs4410790) related to the confounding factors (BMI; previous knee injury; coffee intake) of the knee OA, two other SNPs (rs2472297; rs4410790) related to the confounding factors (coffee intake) of the hip OA. In order to satisfy assumption 2, these SNPs will be eliminated when the corresponding analysis is performed. We used a range of sensitivity analyses to satisfy assumption 3.

### Causal Associations Between Tea Consumption and the Risk of Knee Osteoarthritis

**FIGURE 2:**
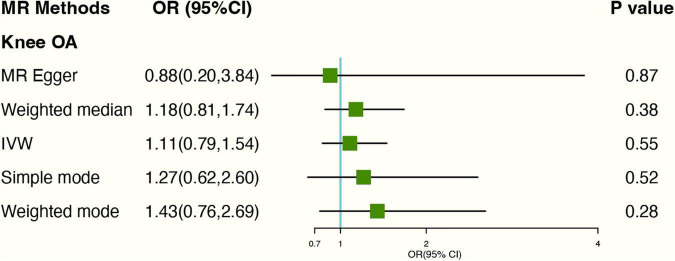
Forest plot of MR study using genetic instruments with knee OA excluding four SNPs (rs11022751; rs1481012; rs2472297; rs4410790); OR, odds ratio; IVW, inverse variance weighted; CI, confidence interval; MR, mendelian randomization; OA, osteoarthritis.

We found four SNPs (rs11022751; rs1481012; rs2472297; rs4410790) related to the confounding factors (BMI; previous knee injury; coffee intake) of the knee OA through the PhenoScanner database (Supplementary Table 1). Weeding out the four SNPs, There was no evidence for the causal association between genetically predicted tea intake and knee OA. The OR was 1.11 (95% CI: 0.79–1.55), using IVW with random effect in MR (Figure 2). The weighted Median methods (OR: 1.18, 95% CI: 0.81,1.74) did not support a causal relationship between tea consumption and the risk of knee OA. The estimates for each SNP and scatter plot for two analyses on knee OA were shown in Supplementary Figures 1, 2.

In MR-Egger regression, the intercept term indicated that horizontal pleiotropy was unlikely to affect the result (Egger intercept: 0.01, *P*-value: 0.77). Additionally, As the funnel plot was visually symmetrical, this indicated that pleiotropy had not been established. SNPs did show slight heterogeneity according to Cochran’s *Q*-test [*Q*-value (df) = 25.08 (13), *P* = 0.03 for MR Egger method; *Q*-value (df) = 25.25 (15), *P* = 0.05 for IVW method]. “Leave-one-out” analysis demonstrated that overall risk estimates were not significantly affected by any single SNP (Supplementary Figure 3).

### Causal Associations Between Tea Consumption and Hip Osteoarthritis

**FIGURE 3:**
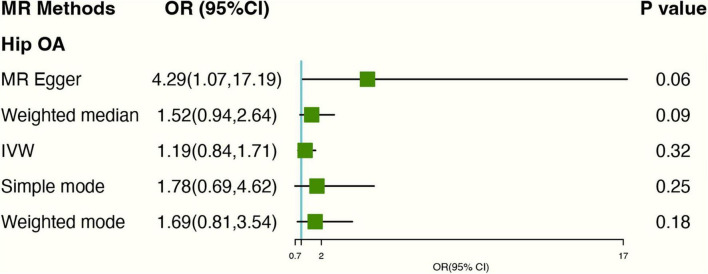
Forest plot of MR study using genetic instruments with hip OA excluding two SNPs (rs2472297; rs4410790). OR, odds ratio; IVW, inverse variance weighted; CI, confidence interval; MR, mendelian randomization; OA, osteoarthritis.

After excluding 2 SNPs (rs2472297; rs4410790) associated with coffee consumption, Based on IVW analyses, no causal relationship was found between tea consumption and hip OA (OR: 1.20, 95% CI: 0.84–1.71) (Figure 3). In MR-Egger regression, the intercept term indicated no horizontal pleiotropy affecting the result (Egger intercept: −0.07, *P*-value: 0.08). As for genetics instruments, Cochran’s Q statistic suggested no heterogeneity: *Q*-value (df) = 16.31 (16), *P* = 0.43 for MR Egger method; *Q*-value (df) = 19.82 (17), *P* = 0.28 for IVW method. The forest and scatter plots can be seen in Supplementary Figures 4, 5 and the leave-one-out plot in Supplementary Figure 6.

### Causal Associations Between Tea Consumption and Rheumatoid Arthritis

**FIGURE 4:**
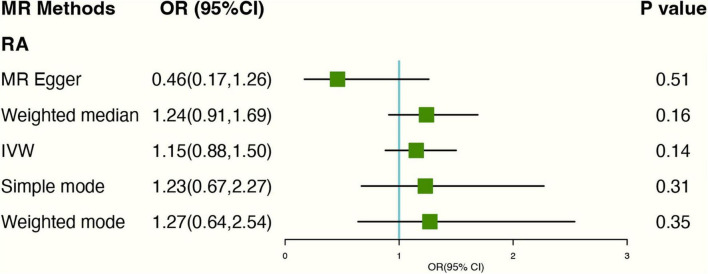
The plot of the MR study uses genetic instruments with RA excluding two SNPs (rs10741694); OR, odds ratio; IVW, inverse variance weighted; CI, confidence interval; MR, mendelian randomization; RA, rheumatoid arthritis.

The PhenoScanner database showed 2 SNPs (rs10741694; rs397074) associated with RA confounding factors. Excluding the two SNPs. Based on genetic instruments using IVW with random effect analyses, there was no causal link between genetically predicted tea consumption and RA (OR: 1.24 95% CI: 0.81–1.91) (Figure 4). The forest plots can be seen in Supplementary Figure 7. The scatter plot for the two analyses was attached as Supplementary Figure 8 and the leave-one-out plot in Supplementary Figure 9.

As for genetics instruments, Cochran’s Q statistic suggested slight heterogeneity: *Q*-value (df) = 20.72 (12), *P* = 0.05 for MR Egger method. Additionally, the intercept of the MR-Egger regression showed no signs of pleiotropy (Egger intercept: 0.05, *P*-value: 0.09).

### Causal Associations Between Tea Consumption and Osteoporosis

**FIGURE 5:**
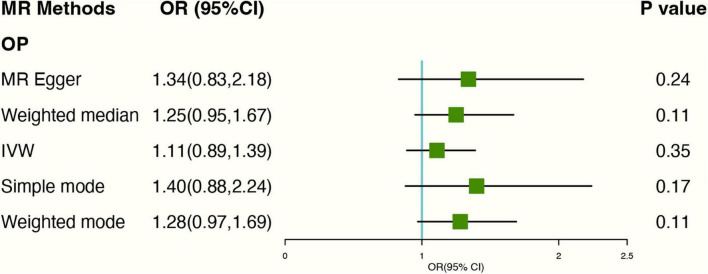
Forest plot of MR study using genetic instruments with OP. OR, odds ratio; IVW, inverse variance weighted; CI, confidence interval; MR, mendelian randomization; OP, osteoporosis.

The risk of OP did not increase with higher levels of tea consumption due to genetic predisposition. The causal ORs for each cup increased in tea consumption were 1.11 (95% CI: 0.89,1.39) in the IVW estimates. At the same time, MR-Egger and Weighted median methods also confirm no causal relationship between tea consumption and OP, and the *P*-value were 0.24 and 0.11, respectively (Figure 5). No heterogeneity found in Cochran’s Q statistic [*Q*-value (df) = 17.47 (15), *P* = 0.29 for MR Egger method; *Q*-value (df) = 18.38 (16), *P* = 0.30 for IVW method]. Detailed forest and scatter plots were shown in Supplementary Figures 10, 11. The leave-one-out analysis shows that none of the single SNP substantially affects the overall risk estimation (Supplementary Figure 12).

## Discussion

As far as we know, This is the first MR study to assess the causation of habitual tea intake with risk of knee OA, hip OA, RA, and OP. Our analysis showed that habitual tea consumption does not contributed to knee OA, hip OA, RA, and OP. There is no sign of reverse causality, selection bias, or weak instruments in this MR study.

The association between tea consumption and risk of knee OA, RA, hip OA, and OP is inconsistent in previous observational studies. According to the study of Katiyar et al., IL-1β plays an important role in inflammatory processes that cause OA and other inflammatory diseases; green tea polyphenols were effective in reducing IL-1β-induced inflammatory cytokines preventing the development of OA (8). The results of the cross-sectional study reported by Jin et al. indicated that higher tea consumption (>750 mL/day) was associated with lower RA disease activity compared with non-tea drinkers (9). Kang et al. drew a meta-analysis including 2 prospective cohort studies, 4 case-control studies, 11 cross-sectional studies (38). They found that the OR of OP for the highest vs. the lowest tea consumption types was 0.62 (38). The subgroup analysis indicated tea consumption reduced OP risk (38). In a study in Iran, bone mineral density at the lumbar spine and hip were measured, and it was found that habitual tea consumption was associated with better bone health in women but not in men (35).

However, a prospective cohort study of The Health Initiative Observational Study discovered that women who drank four or more cups of tea per day had an HR of 1.78 (95% CI, 0.83–3.82) for developing RA compared to those without drinking (15). Researchers from the nationwide Women’s Health Study found that habitual tea drinking had little effect on bone density and did not significantly alter fracture risk among United States postmenopausal women (*n* = 91,465) (16). For the causal relationship between tea intake and OA, RA, and OP, the conclusions of the observational studies were inconsistent, so we designed this MR study.

Several biological mechanisms could underlie the reverse relationship between tea intake and knee OA and RA risk. Firstly, in rat animal models. The tea significantly impacts articular cartilage as well as hyaline cartilage in the growth plate (39). The integrity of fetal articular cartilage may be affected by caffeine exposure in some pregnant women. The caffeine in tea may also increase mechanical stress-induced OA, suggesting that caffeine may be a factor in OA development (40). Secondly, some basic cell lever research reported that Tea contains caffeine and fluoride, which inhibit collagen production, mineralization, osteocalcin levels, and chick osteoblasts which are essential ingredients in bone development (41). Finally, some molecular studies reported that mesenchymal stromal cells treated with caffeine in tea derived from rat bone marrow also showed a similar decrease in ALP expression, RUNX2, and collagen 1 expression, which is very important in cartilage development. In this study, we found that some of the tea-related SNPs (rs2472297; rs4410790) were associated with coffee intake, and to satisfy assumption 2, we excluded these SNPs.

Compared to retrospective analysis and case-control study, the MR method can reduce bias due to individuals. Especially, recall bias was a certainty in an observational study, and the frequency and amount of tea consumption were often inexact in a survey. Additionally, data collection and analysis can be very costly. The MR method reduces this issue to an extent, and sensitivity analyses can be improved by sequentially applying various algorithms.

The two-sample MR design is the greatest strength of our study; Firstly, this study had a large sample size with Summarizing genetic data, which could overcome limitations of conventional epidemiological study designs, such as confounding and reverse causality. It is more time-efficient and less expensive than RCT. Secondly, the MR-PRSSO method was used to identify outliers and demonstrate pleiotropy. Thirdly, we used the “leave-one-out” method as sensitivity analyses increasing the robustness of conclusions.

There were some limitations in this study. First, to minimize the possible impact of stratified populations, the samples of European ancestry were limited. Hence, we could not generalize our results to other ethnicities. Second, the brewing method and type of tea were unclear because there was no specific information about them. The effects of tea type and brewing methods were not assessed. Finally, The data used in this study are summary statistics data, so there is no individual information, and it is impossible to accurately calculate the sample overlap between exposure and outcome.

## Conclusion

According to our study, there was no statistical power to confirm a causal relationship between tea consumption and the risk of knee OA, hip OA, RA, and OP.

## Data Availability Statement

The original contributions presented in the study are included in the article/Supplementary Material, further inquiries can be directed to the corresponding author.

## Author Contributions

SoC and YC participated in study design. SoC, DH, and YP acquired and analyzed the data. ShC participated in the study’s supervision. SoC drafted the manuscript. All authors contributed to the article and approved the submitted version.

## Conflict of Interest

The authors declare that the research was conducted in the absence of any commercial or financial relationships that could be construed as a potential conflict of interest.

## Publisher’s Note

All claims expressed in this article are solely those of the authors and do not necessarily represent those of their affiliated organizations, or those of the publisher, the editors and the reviewers. Any product that may be evaluated in this article, or claim that may be made by its manufacturer, is not guaranteed or endorsed by the publisher.

## Footnotes

1
http://www.nealelab.is/uk-biobank

2
https://www.finngen.fi

3
https://gwas.mrcieu.ac.uk/
