# Supplementary material for: Causal Association Between Tea Consumption and Bone Health: A Mendelian Randomization Study
Source: Front Nutr. 2022 Apr 26;9:872451. doi: 10.3389/fnut.2022.872451 (PMC9087269; doi:10.3389/fnut.2022.872451)
Supplement: Supplementary file 1 [file Data_Sheet_1.docx]

Supplementary Material

**1、Supplementary Tables**

**Table 1S.** Association of tea consumption-associated SNPs with

established risk factors for Knee OA, Hip OA, RF, OS.

|  | **Knee OA** | | | | **Hip OA** | | | **RF** | | | **OS** | | |
| --- | --- | --- | --- | --- | --- | --- | --- | --- | --- | --- | --- | --- | --- |
| **SNP** | **BMI** | **previous knee injury** | **Gout** | **coffee intake** | **Hip dysplasia** | **Glucocorticoid use** | **coffee intake** | **hypertension** | **diabetes** | **smoking** | **vitamin D intake** | **Glucocorticoid use** | **smoking** |
| rs2315024 | - | - | - | - | - | - | - | - | - | - | - | - | - |
| rs2465018 | - | - | - | - | - | - | - | - | - | - | - | - | - |
| rs9624470 | - | - | - | - | - | - | - | - | - | - | - | - | - |
| rs10741694 | - | - | - | - | - | - | - | 1.18E-11 | - | - | - | - | - |
| rs11022751 | 4.19E-09 | - | - | - | - | - | - | - | - | - | - | - | - |
| rs11487328 | - | - | - | - | - | - | - | - | - | - | - | - | - |
| rs4410790 | - | - | - | 1.40e-29 | - | - | 1.40e-29 | - | - | - | - | - | - |
| rs4817505 | - | - | - | - | - | - | - | - | - | - | - | - | - |
| rs1481012 | - | - | 2.05E-32 |  | - | - | - | - | - | - | - | - | - |
| rs1601409 | - | - | - | - | - | - | - | - | - | - | - | - | - |
| rs1669433 | - | - | - | - | - | - | - | - | - | - | - | - | - |
| rs199621380 | - | - | - | - | - | - | - | - | - | - | - | - | - |
| rs12591786 | - | - | - | - | - | - | - | - | - | - | - | - | - |
| rs12600469 | - | - | - | - | - | - | - | - | - | - | - | - | - |
| rs140775622 | - | - | - | - | - |  | - | - | - | - | - | - | - |
| rs149375687 | - | - | - | - | - |  | - | - | - | - | - | - | - |
| rs2472297 | - | - | - | 2.00e-24 | - |  | 2.00e-24 | - | - | - | - | - | - |
| rs3815455 | - | - | - | - | - |  | - | - | - | - | - | - | - |
| rs6697410 | - | - | - | - | - |  | - | - | - | - | - | - | - |
| rs73424602 | - | - | - | - | - |  | - | - | - | - | - | - | - |
| rs73073176 | - | - | - | - | - |  | - | - | - | - | - | - | - |
| rs7999399 | - | - | - | - | - |  | - | - | - | - | - | - | - |

The reported P value were obtained from PhenoScanner v2 on November 12, 2021. BMI：Body Mass Index  RF: Rheumatoid Arthritis. OA: Osteoarthritis. OP: Osteoporosis.

**2 Supplementary Figure**

**Figure 1S**. The forest plot for the causal effects of tea consumption-associated SNPs on knee OA

**Figure 2S.** Scatter plot of genetic associations comparing tea consumption to the genetic associations with knee OA;

**Figure 3S.** Leave-one-out sensitivity analysis for knee OA using primary genetic instruments;

**Figure 4S.** The forest plot for the causal effects of tea consumption-associated SNPs on hip OA;

**Figure 5S**. Scatter plot of genetic associations comparing tea consumption to the genetic associations with hip OA;

**Figure 6S**. Leave-one-out sensitivity analysis for the hip OA;

**Figure 7S**. The forest plot for the causal effects of tea consumption-associated SNPs on RF;

**Figure 8S**. Scatter plot of genetic associations comparing tea consumption to the genetic associations with RF;

**Figure 9S**. Leave-one-out sensitivity analysis for the RF;

**Figure 10S**. The forest plot for the causal effects of tea consumption-associated SNPs on OP;

**Figure 11S**. Scatter plot of genetic associations comparing tea consumption to the genetic associations with OP;

**Figure 12S**.Leave-one-out sensitivity analysis for the OP;
